# SERS Microsensors for the Study of pH Regulation in Cystic Fibrosis Patient-Derived Airway Cultures

**DOI:** 10.1021/acssensors.4c00279

**Published:** 2024-04-25

**Authors:** William H. Skinner, Nicola Robinson, Gareth R. Hardisty, Robert D. Gray, Colin J. Campbell

**Affiliations:** † EaStCHEM School of Chemistry, The University of Edinburgh, King’s Buildings, Mayfield Road, Edinburgh EH9 3FJ, U.K.; ‡ Centre for Inflammation Research, The Queen’s Medical Research Institute, The University of Edinburgh, 47 Little France Crescent, Edinburgh EH16 4TJ, U.K.; § School of Infection and Immunity, University of Glasgow, Sir Graeme Davies Building, University Place G12 8QQ, Scotland

## Abstract

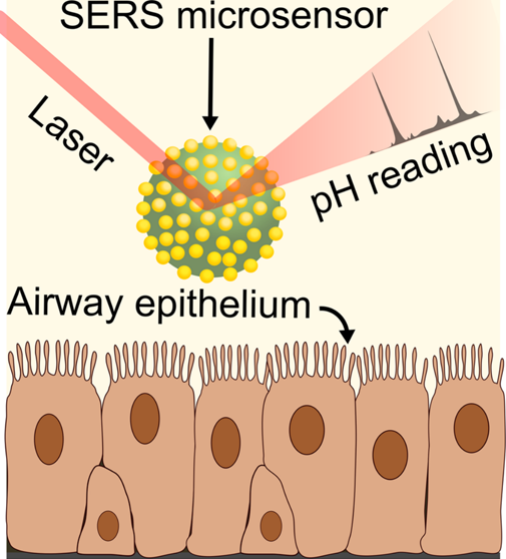

Acidification of the airway surface liquid in the respiratory system could play a role in the pathology of Cystic Fibrosis, but its low volume and proximity to the airway epithelium make it a challenging biological environment in which to noninvasively collect pH measurements. To address this challenge, we explored surface enhanced Raman scattering microsensors (SERS-MS), with a 4-mercaptobenzoic acid (MBA) pH reporter molecule, as pH sensors for the airway surface liquid of patient-derived in vitro models of the human airway. Using air–liquid interface (ALI) cultures to model the respiratory epithelium, we show that SERS-MS facilitates the optical measurement of trans-epithelial pH gradients between the airway surface liquid and the basolateral culture medium. SERS-MS also enabled the successful quantification of pH changes in the airway surface liquid following stimulation of the Cystic Fibrosis transmembrane conductance regulator (CFTR, the apical ion channel that is dysfunctional in Cystic Fibrosis airways). Finally, the influence of CFTR mutations on baseline airway surface liquid pH was explored by using SERS-MS to measure the pH in ALIs grown from Cystic Fibrosis and non-Cystic Fibrosis donors.

Surface enhanced Raman scattering (SERS) pH sensors have been extensively applied to intracellular sensing with a primary focus on measuring pH in the endosomal transport chain.^1−3^ The development of physiologically mimetic tissue models such as air–liquid interfaces (ALIs) and organoids has brought into focus the importance of the extracellular physical and chemical microenvironment and the unique requirements of sensors needed to monitor these niches.^4,5^

SERS substrates for extracellular pH sensing have been fabricated from nanoparticle assemblies on planar surfaces, electrospun nanofibers, and photolithographic substrates.^6−8^ Sensing with these substrates requires the direct culture of cells on the substrate surface, limiting pH measurements to the basolateral extracellular environment and preventing their application to more complex three-dimensional (3D) cell culture platforms.

Recently, we demonstrated SERS microsensors (SERS-MS) as powerful tools to probe pH in different extracellular microenvironments within 3D airway organoid cultures grown from primary human cells.^9^ SERS-MS were mixed into the extracellular matrix of organoid cultures and engulfed by the growing organoids over a culture period of 2 weeks. This method enabled the first microinjection-free measurement of organoid lumen pH but had inherently low throughput and required optimization of growth conditions to ensure SERS-MS uptake by organoids. In this paper, we push SERS-MS pH sensing further with a higher-throughput application in ALIs, the lab standard for in vitro airway models. Our assay requires no long-term sensor incubation period or alterations to established cell culture protocols; the SERS-MS is simply pipetted onto the apical surface of the cells prior to spectral acquisition. The current work demonstrates the potential of SERS-MS to monitor trans-epithelial ion-channel dynamics in a microenvironmental niche relevant to the genetic disease Cystic Fibrosis.

SERS-MS have a diameter of 20 μm, making them large enough to avoid endocytosis but small enough to probe extracellular microscale environments.^10^ SERS-MS are functionalized with pH-sensitive thiol 4-mercaptobenzoic acid (MBA) and report on local pH using the relative intensity of the carboxylate vibration *v*_s_(COO), which reflects the protonation state of the surface-bound MBA population. These properties make SERS-MS excellent candidates for probing pH in the airway surface liquid of the respiratory epithelium.

The airway surface is modeled in vitro with ALI cultures that recapitulate key characteristics of the in vivo airway epithelium such as pseudostratification, tight junction expression, and heterogeneous cell populations containing ciliated and mucous-secreting cells.^11,12^ ALIs are ideal for studies of changes in airway chemistry associated with the life-limiting autosomal recessive genetic disease Cystic Fibrosis, a symptom of which is chronic infection and inflammation of the lungs and airways.^13−16^

Cystic Fibrosis is caused by a mutation in the gene encoding the Cystic Fibrosis transmembrane conductance regulator (CFTR) protein, which transports chloride and bicarbonate ions to the airway surface liquid.^17^ The transport of pH buffer bicarbonate has garnered much interest because acidified airway surface liquid has been shown to reduce the activity of antimicrobial peptides and increase the risk of airway infection.^5^ However, whether the airway surface liquid is more acidic in individuals with Cystic Fibrosis is still debated.^13,18^

The airway surface liquid is a challenging biological environment in which to measure pH by using conventional pH electrodes and fluorescent probes. pH measurements with electrodes can disrupt the underlying cell layer, and to avoid this, the airway surface liquid is often diluted with a low buffering capacity solution to increase its volume.^16^ Fluorescence probes have been used to measure airway surface liquid pH in situ; however, these probes are sensitive to photobleaching.^18,19^ SERS offers an alternative pH sensing technique that allows pH measurements in close proximity to the apical surface of epithelial cells and the basolateral cell culture medium in a single imaging run (Figure 1). This approach could provide a more holistic picture of pH within individual in vitro epithelial cell models and a deeper understanding of the role of pH in the pathophysiology of Cystic Fibrosis.

**Figure 1:**
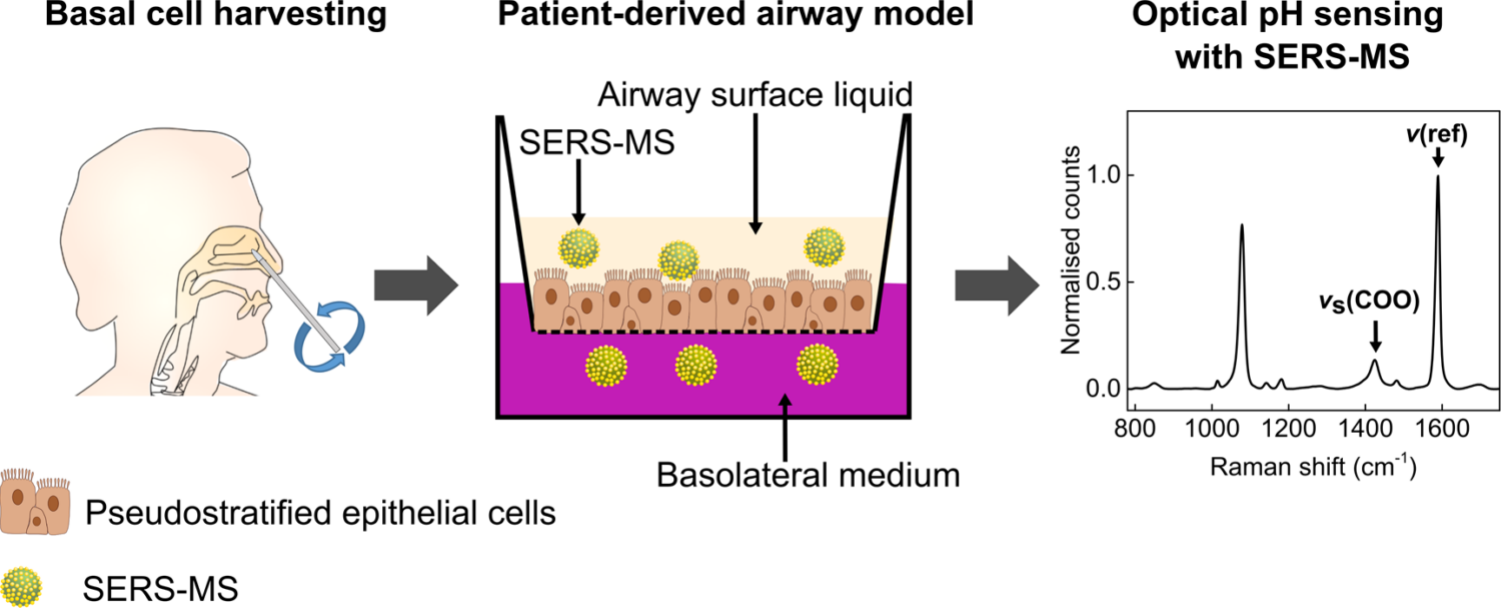
Schematic of an in vitro airway surface liquid pH sensing experiment with SERS-MS. Basal epithelial cells were collected from the inferior nasal turbinate of volunteers with and without Cystic Fibrosis. These cells were cultured as ALIs and differentiated into epithelial cells after 21 days. SERS-MS were then added to the airway surface liquid, and SERS spectra were collected to measure local pH optically. Ratiometric pH sensing was conducted using the spectral peaks highlighted as *v*_s_(COO) and *v*(ref).

In this paper, we explore the direct delivery of SERS-MS to the apical and basolateral environments of ALI cultures to measure the trans-epithelial pH and dynamic pH events associated with the activation of the CFTR ion channel. We then compare the airway surface liquid pH measured using SERS-MS in ALIs grown from Cystic Fibrosis and non-Cystic Fibrosis human donors to explore whether CFTR mutations result in abnormal pH regulation.

## Materials and Methods

### Cell Harvesting

Ethical approval was gained (North of Scotland Research Ethics Committee, IRAS ID 286836), and participants were recruited and consented. Cells were obtained by nasal brushing. The inferior nasal turbinate was visualized with an otoscope and 9 mm specula attachment. A sterile cytology brush was passed through the operating channel to brush under direct vision. The brush was transferred into a 15 mL Falcon tube containing 5 mL of RMPI medium (Gibco) with 1% Penicillin/Streptomycin, 10% fetal calf serum, and 1% L glutamine.

### Basal Cell Expansion

The Falcon tubes were vortexed before removing the brush and then centrifuged at 300*g* for 5 min at 20 °C. The supernatant was discarded, and the cell pellet was suspended in 10 mL of Pneumacult Ex-Plus (StemCell Technologies) basal cell expansion medium with supplements (Table S1). The suspension was transferred to a T75 tissue culture flask (Corning) which was precoated with matrix proteins from 804G conditioned medium. The medium was changed every 48–72 h and passaged at 90% confluence using TrypLE Express (Gibco).

### Air–Liquid Interface (ALI) Culture

ALIs were grown on 24-well Tissue Culture (TC) Inserts (Sarstedt, 83.3932.041) with a pore size of 0.4 μm and a pore density of 2 × 10^6^ pores/cm^2^ (Figure S1). The TC inserts were precoated with matrix proteins from 804G conditioned medium, and the apical compartment of each was seeded with 100,000 basal cells in 300 μL of basal cell expansion medium (Table S1). Basal cell medium (800 μL) was also added to the basolateral compartments of the wells. The cells were incubated at 37 °C and 5% CO_2_ for 24–48 h until a confluent layer had formed. The culture medium was then removed from the apical and basolateral compartments, and 500 μL of ALI medium (Table S2) was added to the basolateral compartment of the wells while the apical compartment was left empty. This step is referred to as the air-lift. The cells were incubated for a further 21 days (medium refreshed every 48–72 h) following the air-lift to achieve differentiation into a pseudostratified epithelium.

### Fluorescence Staining and Imaging

The ALIs were fixed with 4% paraformaldehyde for 30 min and then washed with 70% ethanol (EtOH) in distilled water followed by 2 washes with phosphate buffered saline (PBS). The cells were permeabilized with Triton ×100 (0.1% in PBS) for 15 min and then washed twice with PBS. A drop of proteinase K was added to 1 mL of 0.1% Tween 20 in PBS and then added to the ALI for 30 min at 37 °C. The ALIs were washed twice with PBS and blocked with 25% goat serum in PBS for 1 h at room temperature followed by a PBS wash. Cilia were stained with anti-α tubulin (mouse, 1:200) in 10% goat serum PBS for 1 h at 37 °C then washed twice with PBS. The next day, Alexa Fluor 488 (goat antimouse, 1:500) in 10% goat serum in PBS was added to the ALIs for 1 h at room temperature and shielded from light. The ALIs were then washed twice with PBS and the nuclei stained with Hoescht (1:1000 in 10% goat serum in PBS) and washed twice with PBS. The stained ALIs were imaged with a Leica TCS SP8 confocal microscope using a 20× objective (Leica, HC PL APO 20×/0.75 CS2). Hoescht was imaged using a 405 nm diode laser with emission detection set to 410–516 nm. For Alexa Fluor 488, an argon laser was used with emission detection set to 555–625 nm. The z-step interval was 0.69 μm. The resulting images were analyzed in ImageJ with the Bio-Formats plugin.^20,21^

### SERS Microsensors (SERS-MS) Fabrication

SERS-MS were fabricated using 20 μm TentaGel M NH_2_ microspheres (Rapp Polymere GmbH, M30202, 2.4 × 10^8^ particles/g) as previously described.^9^ TentaGel M NH_2_ powder (1 mg) was added to 3 mL of citrate-capped 150 nm gold nanoparticle colloid (Sigma-Aldrich, 746649), sonicated for 10 min, and stored at 4 °C. The day before sensing experiments, the particles were pelleted via centrifugation and suspended in a 100 μM solution of 4-mercaptobenzoic acid (MBA) (Sigma-Aldrich, 662534) in 1% EtOH (≥99.8%, Sigma-Aldrich) in deionized (DI) water. The MBA solution was prepared by dissolving MBA in EtOH at 10 mM and diluting 1:100 with DI water. The SERS-MS were stored in the MBA solution overnight at 4 °C before washing with 70% EtOH in DI water and twice with sterile saline solution. The SERS-MS were suspended at a final concentration of 2000 SERS-MS/μL in sterile saline before application to ALI cultures. For scanning electron microscope (SEM) imaging, SERS-MS was suspended in EtOH and dried onto an aluminum stub. SEM images were collected with a Zeiss Crossbeam 550.

### SERS-MS pH Calibration

All spectra were collected on a Renishaw InVia Raman microscope with a water immersion objective (Olympus, LUMPlanFL N 60×/1.00, working distance 2 mm) and a 785 nm laser. Leibovitz’s L-15 cell culture medium (Thermo Fisher, 11415064) containing 10% fetal bovine serum was adjusted to pH 5.80, 6.20, 6.63, 7.00, 7.45, and 7.80 with KH_2_PO_4_ solution (0.1 M) and pH measured with an Orion 9110DJWP double-junction pH electrode (accuracy ±0.02 pH units). A 100 μL droplet of each pH buffer was pipetted between the immersion objective and a CaF_2_ slide, and 1 μL of SERS-MS suspension (2000 SERS-MS/μL in saline solution) was pipetted directly into the droplet of the medium. The SERS-MS sank to the bottom of the droplet and rested on the CaF_2_ slide, at which point SERS spectra were collected with 0.033 mW laser power and a 1 s acquisition time. Six point spectra were collected from different locations on individual SERS-MS and 10 SERS-MS were analyzed at each pH. All spectra were smoothed with 9-point Savitsky-Golay smoothing (polynomial order 3) and the background subtracted with WiRE Intelligent Fitting baseline subtraction. The maximum intensity of the MBA carboxylate peak (*v*_s_(COO)) at ∼1400 cm^–1^ and the *v*_8a_ ring breathing mode (*v* (ref)) at ∼1590 cm^–1^ was extracted from each spectrum in MATLAB. Only spectra with *v*(ref) > 1000 counts were taken forward for pH measurements. Origin 2021b was used to plot the calibration data and fit a Boltzmann curve (eq S1 and Table S3).

### SERS-MS pH Validation

SERS-MS pH sensing in the airway surface liquid of ALIs was validated by creating an airway surface liquid of known pH from a buffered L-15 medium on the surface of fixed ALIs. Fixed ALIs were soaked in L-15 medium at pH 5.93, 6.48, or 6.95 for 30 min, then, all but a thin layer of buffer solution was removed from the apical compartment to simulate the airway surface liquid. SERS-MS suspension (1 μL) was then added to the apical surface of the fixed cells. For Raman imaging, the TC insert was inverted and a 100 μL droplet of buffered L-15 solution was pipetted between the objective and the basolateral side of the TC insert (Figure S2). Spectra were collected by focusing through the TC insert membrane on individual SERS-MS on the apical surface of fixed epithelial cells and using 0.033 mW laser power and 1 s acquisition time. Spectra were processed as outlined above and a pH value was calculated for each spectrum using eq S1.

### Trans-Epithelial pH Measurements

Airway surface liquid pH measurements were taken in 21-day-old ALI cultures a day after refreshing the medium in the basolateral compartment. All measurements were collected from live ALI cultures at 5% CO_2_. SERS-MS suspension (1 μL) was added directly to the apical surface of the ALI. The ALI was then inverted, and a 100 μL droplet of the basolateral medium was pipetted between the immersion objective and the basolateral side of the TC insert (Figure S3). SERS-MS suspension (1 μL) was then added to the droplet of basolateral medium. Spectra were collected from ≥6 SERS-MS particles at different locations in the airway surface liquid (4–5 spectra per SERS-MS to capture the average pH environment of an individual SERS-MS), then, spectral acquisition was repeated for SERS-MS in the basolateral medium by adjusting the microscope z-height. Trans-epithelial pH measurements were conducted in three ALIs grown from different donors with Cystic Fibrosis.

### Trans-Epithelial Electrical Resistance (TEER) Measurement

An EVOM voltohmmeter was used to measure the TEER of ALI cultures. Fresh medium (200 μL) was added to the apical compartment and 500 μL to the basolateral compartment of the TC insert. The EVOM “chopstick” electrodes were lowered into the apical and basal compartments to measure the resistance across the ALI. TEER was calculated with eq S2.

### Airway Surface Liquid pH Following CFTR Activation

Three non-Cystic Fibrosis donors were recruited, and 2 ALIs were cultured from each. After 21 days of ALI culture and 1 day after refreshing the medium in the basolateral compartment, 1 μL of SERS-MS suspension was added to the apical surface of each ALI, and SERS spectra were collected as described previously. Forskolin and 3-isobutyl-1-methylxanthine (IBMX) were then added to the basolateral medium of one ALI from each donor at a concentration of 10 and 100 μM, respectively. Stock solutions of Forskolin and IBMX were prepared in DMSO, and hence DMSO was added as a control to the basolateral medium of the second ALI from each donor at 0.2% volume. After 2 h of incubation at 37 °C and 5% CO_2_, spectra were again collected from SERS-MS in the airway surface liquid of Forskolin/IBMX and control-treated ALIs.

### Airway Surface Liquid pH in Cystic Fibrosis and Non-Cystic Fibrosis ALIs

Basal epithelial cells were harvested from 6 Cystic Fibrosis and 6 non-Cystic Fibrosis donors, and ALIs from each were cultured for 21 days. The cell culture medium in each ALI was refreshed 1 day prior to pH measurements. SERS-MS were added to the airway surface liquid and spectra for pH measurements were collected under the conditions described above.

## Results and Discussion

### Air–Liquid Interface (ALI) Culture

**Figure 2:**
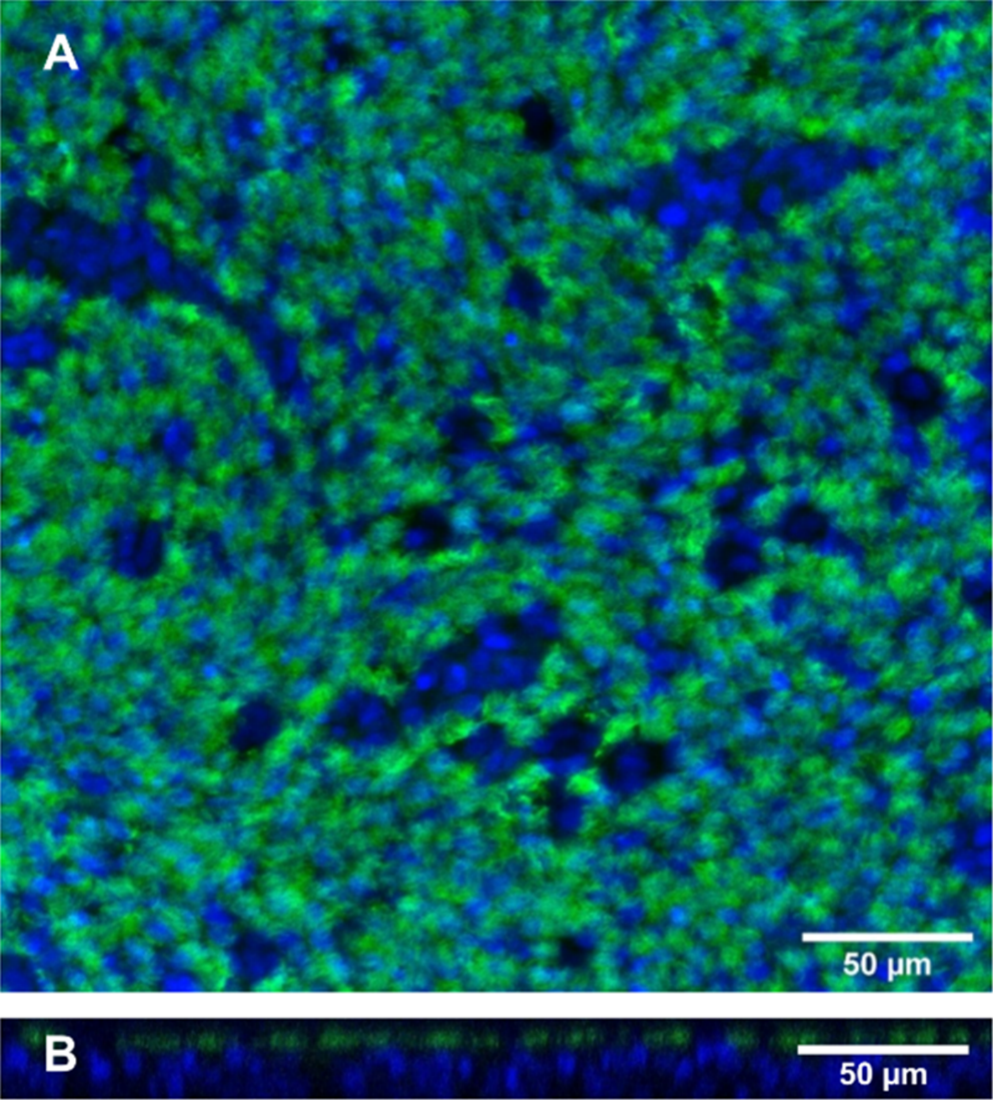
(A) *z*-Stack projection of an ALI stained for cell nuclei (Hoescht, blue) and cilia (anti-α tubulin, green). (B) Cross section of the *z*-stack in (A) showing epithelial cell pseudostratification and cilia expression on the apical surface (cell polarization).

**Figure 3:**
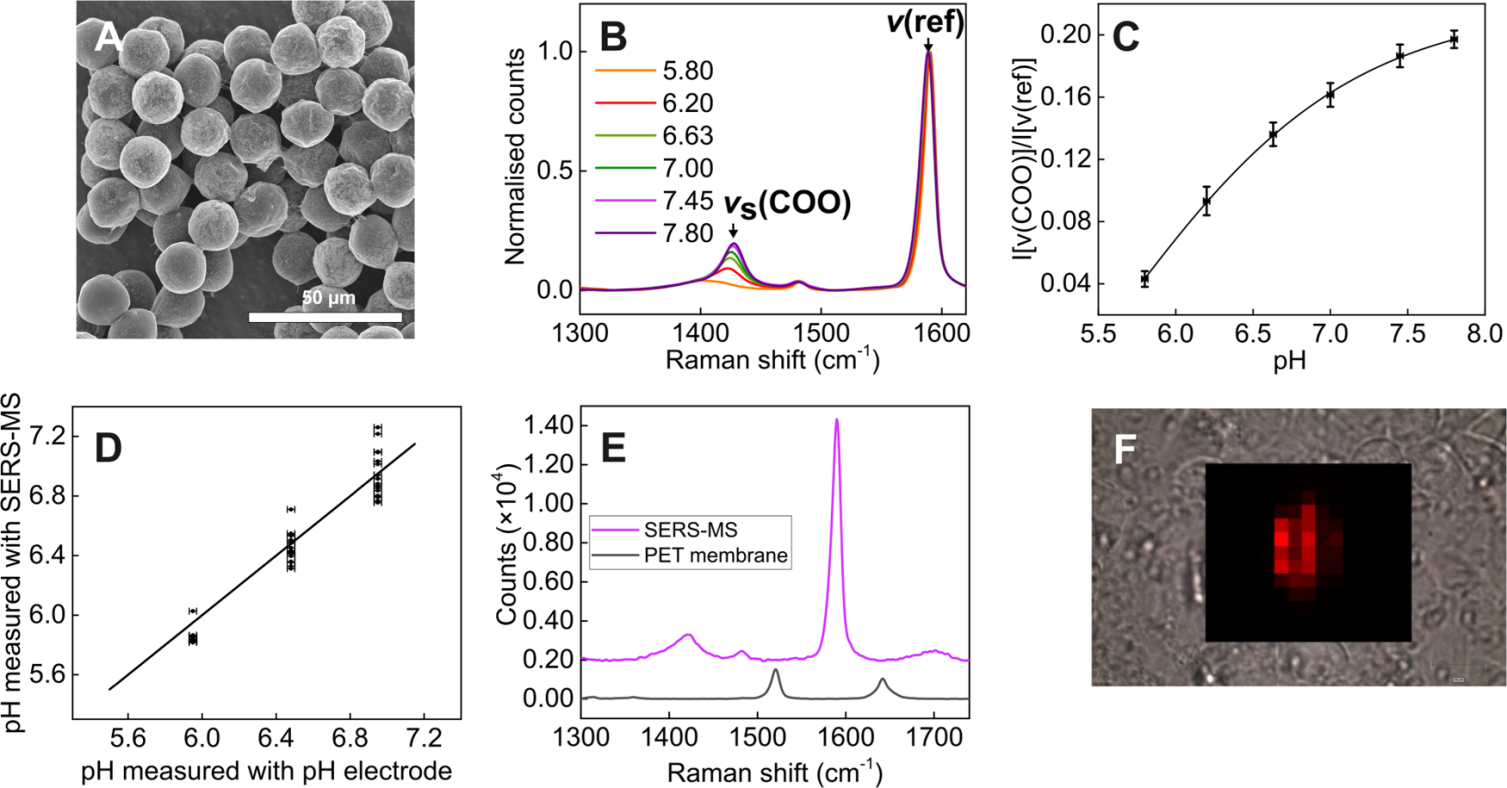
(A) SEM image of SERS-MS. (B) Mean SERS-MS spectra collected in a pH-adjusted cell culture medium. Spectra were normalized to *v*(ref) (complete spectra are presented in Figure S4). (C) SERS-MS calibration curve in a pH-adjusted cell culture medium. The intensity ratio of *v*_s_(COO) and *v*(ref) are plotted against pH. Data points are the mean ratio from spectra collected at each pH, *y*-axis error bars represent the SD, and *x*-axis error bars the precision of the pH meter, the solid line is a Boltzmann curve fit. (D) pH measured using SERS-MS in pH-adjusted culture medium on the apical surface of fixed ALIs compared to the pH measured with a pH electrode in 1 mL of the same medium. *X*-axis error bars represent the precision of the pH meter. The black line represents the SERS-MS pH = electrode pH. RMSE = 0.12 for SERS-MS pH vs electrode pH. The average pH measured from 18 spectra at each pH was 5.85 ± 0.05, 6.47 ± 0.10, and 6.94 ± 0.14. (E) SERS-MS spectrum collected from the apical surface of a fixed ALI in cell culture medium adjusted to pH 6.48 (purple) (0.033 mW laser power and 1 s acquisition time) and the spectrum of the polyethylene terephthalate membrane of the TC insert (black) (0.165 mW laser power and 1 s acquisition time). Both spectra were smoothed and baseline corrected. The SERS-MS spectrum is offset by 2000 counts on the *y*-axis for clarity. (F) Raman map of a SERS-MS lying on the apical surface of an ALI with heatmap showing intensity of MBA’s *v*_8a_ ring breathing mode (*v*(ref)) at ∼1590 cm^–1^ localized to the SERS-MS (brightest pixel represents 9200 counts).

Basal cells were cultured as outlined above and after 21 days formed a pseudostratified epithelium containing polarized cells expressing cilia on their apical facet (Figure 2A,B). Cell polarization and pseudostratification are both fundamental features of the in vivo epithelium. This confirmed our ALIs as valid models in which to develop SERS-MS (Figure 3A) for pH sensing in the airway surface liquid and to explore the role of airway pH in the pathophysiology of Cystic Fibrosis.

### SERS-MS Fabrication, Calibration, and Validation

We fabricated SERS-MS by assembling gold nanoparticles onto the surface of commercially available amino TentaGel microspheres (20 μm) via gold-amine interactions and functionalized the gold nanoparticles with MBA, via gold–thiol bonding.^9^ The resulting SERS-MS are SERS-active microparticles and can be delivered to the extracellular environment of tissue cultures. SERS-MS are restricted to the extracellular environment by their size; 20 μm is similar in size to a typical human cell and unlikely to be endocytosed.^8^ However, the microparticle nature of SERS-MS means that they can be delivered to the apical and basolateral environments of ALI cultures to probe pH gradients that form across the epithelial barrier in vitro.

SERS-MS use the spectral changes associated with the protonation state of MBA to measure pH optically.^22,23^ SERS-MS were calibrated in pH-adjusted cell culture medium at pH 5.80–7.80, this range encompasses the airway surface liquid pH range reported in the literature for airway ALIs.^13,18^ A calibration curve was created to calculate local pH from the SERS-MS spectrum by plotting the intensity ratio of *v*_s_(COO) and *v*(ref) and fitting a Boltzmann curve (Figure 3B,C). The slope of the calibration curve here is more gradual than we reported previously for sensing in 3D hydrogel organoid cultures because hydrogel encapsulation influences the sensitivity of SERS pH sensors.^24^

To test the accuracy of our sensor in the airway surface liquid environment, we added SERS-MS and pH-adjusted culture medium to the apical surface of fixed ALIs to simulate the airway surface liquid and measured pH optically (Figure 3D,F). The resolution of our SERS pH measurement decreased slightly at higher pH values (Figure 3D), and this spreading of data points at higher pH values is driven by the shallower gradient in the calibration curve at higher pH values (Figure 3C). An RMSE value of 0.12 was achieved between the pH predicted with SERS-MS on the apical surface of ALIs and the pH measured with a pH electrode in a larger volume of pH-adjusted cell culture medium. This RMSE is similar to previously reported values for MBA SERS pH sensing and confirms our approach to measuring pH on the apical surface of ALIs does not reduce the accuracy of MBA-based SERS pH sensors.^25^ This confirmed SERS-MS as a valuable tool to measure pH in low-volume environments not easily accessible with pH electrodes, such as in close proximity to the apical surface of epithelial cells in the airway surface liquid of ALIs.

### Measuring Trans-Epithelial pH Gradients with SERS-MS

**Figure 4:**
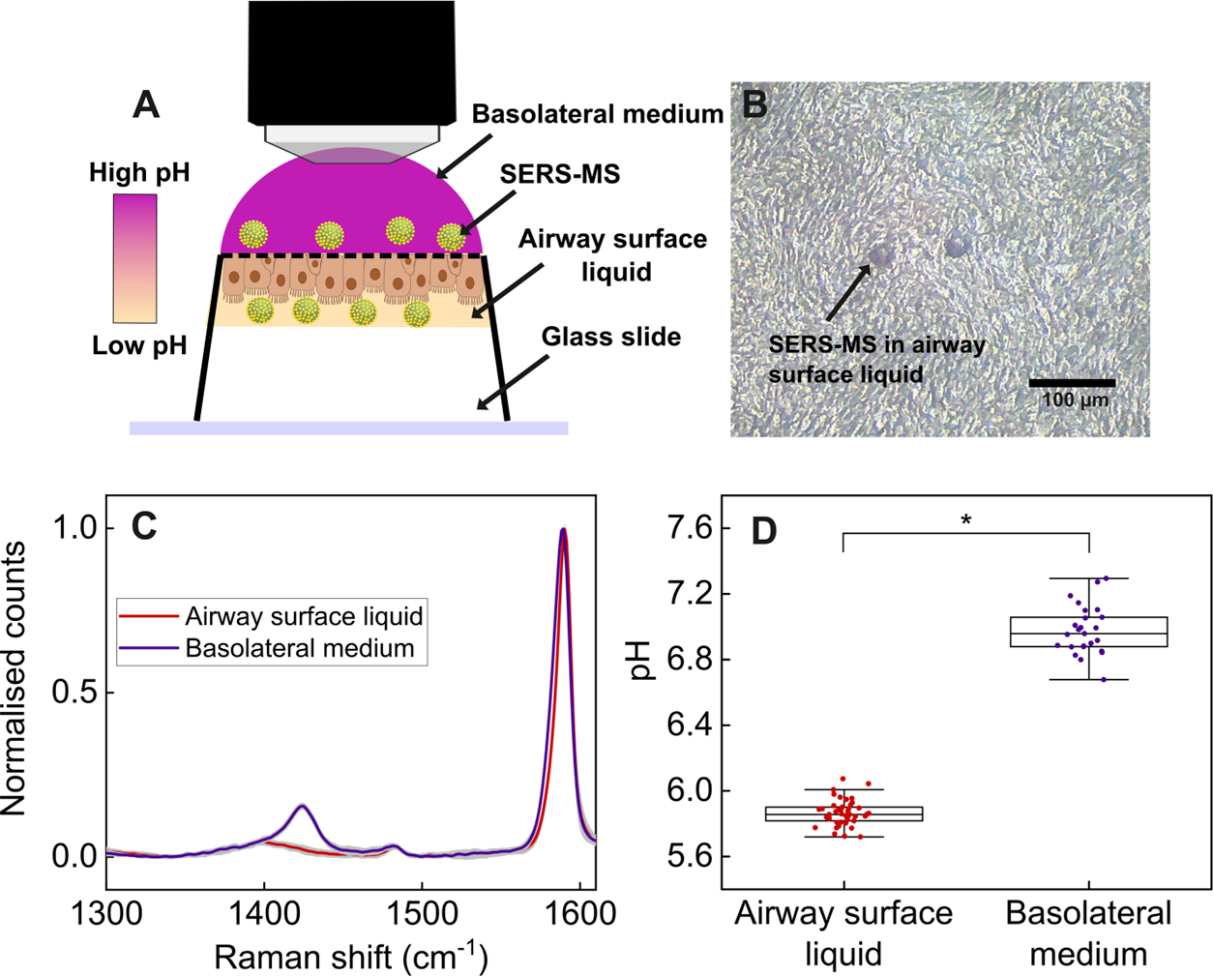
(A) Schematic of spectral collection from SERS-MS in the airway surface liquid and basolateral medium of an ALI (not to scale). The tissue culture insert was inverted to allow a water immersion lens to be positioned on the basolateral side of the ALI. Surface tension kept the low-volume airway surface liquid in place on the apical surface. (B) Bright-field image of SERS-MS lying on the apical surface of epithelial cells in an ALI (Video available in the Supporting Information). (C) Mean normalized SERS-MS spectra collected from the airway surface liquid and basolateral medium of a Cystic Fibrosis ALI. The gray shaded area indicates the standard deviation of the spectra. (D) Airway surface liquid and basolateral pH calculated from SERS-MS spectra in (C). Each data point represents the pH calculated from a single spectrum using the calibration curve in Figure 3. The center line of the box plot is the median, bottom and top of the box show the 25th and 75th quantiles, respectively, and whiskers extend to 1.5 times the interquartile range; * indicates significant pH difference by *t* test (*p* < 0.05).

Epithelial cells in the respiratory tract maintain a barrier between the airway lumen and internal tissues. In ALIs, epithelial cells replicate this barrier function by forming tight junctions between adjacent cells creating a semipermeable size- and ion-specific barrier between the apical and basolateral compartments.^26^ SERS-MS enables the measurement of pH in both low-volume (airway surface liquid) and high-volume (basolateral medium) environments. Figure 4A illustrates the application of SERS-MS to measure the pH in the airway surface liquid and basolateral compartment of ALIs grown from donors with Cystic Fibrosis to determine if tight junctions between epithelial cells limit the flux of HCO_3_^–^ to the airway surface liquid.

Adding SERS-MS to the apical surface of ALIs resulted in the sensors sitting immediately above epithelial cells (Figure 4B) where they were gently buffeted by beating cilia (see the Video in the Supporting Information). By focusing the objective lens on SERS-MS in the airway surface liquid and basolateral medium of an ALI, we collected spectra from SERS-MS in both environments in a single imaging run. Figure 4C presents the mean normalized SERS-MS spectrum collected from the airway surface liquid and basolateral compartment of a Cystic Fibrosis ALI. The relative intensity of the *v*_s_(COO) peak is larger when SERS-MS are in the basolateral compartment, indicating a high proportion of MBA molecules are deprotonated and hence the basolateral medium is more alkaline than the airway surface liquid. The mean airway surface liquid pH calculated from SERS-MS in this ALI was 5.9 (Figure 4d), which is similar to the pH measured by Schultz et al. with a fluorescence-based fiber-optic probe in ALIs derived from lower airway brushings of children with Cystic Fibrosis.^13^

Apical SERS-MS was used for local measurement in a low-volume nonbuffered matrix (the airway surface liquid), and SERS-MS on the basolateral side was used for measurement in a bulk buffered environment (the cell culture medium). The mean pH of the basolateral medium was determined to be 7.0. This difference of 1.1 pH units across the ALI epithelial layer indicates that tight junctions between adjacent cells restrict the flux of HCO^3–^ between the cell culture medium and the airway surface liquid allowing the metabolic activity of the cells to acidify the apical environment. The formation of an acidic apical environment relative to the cell culture medium was confirmed in 2 additional ALIs grown from different Cystic Fibrosis donors (Figure S5). Following spectral acquisition, the mean trans-epithelial electrical resistance (TEER) of the ALIs (a measure of the strength of the tight junctions formed by epithelial cells) was 363 ± 95 Ω·cm^2^, confirming the barrier function of the epithelial layer in ALIs remained intact after spectral acquisition.^27^ These findings suggest that the apical/basal pH gradient may be a measure of tight junction function at the baseline, which could be a useful additional measurement when assessing the integrity of epithelial ALI cultures.

### Airway Surface Liquid pH Measurements Following CFTR Stimulation

**Figure 5:**
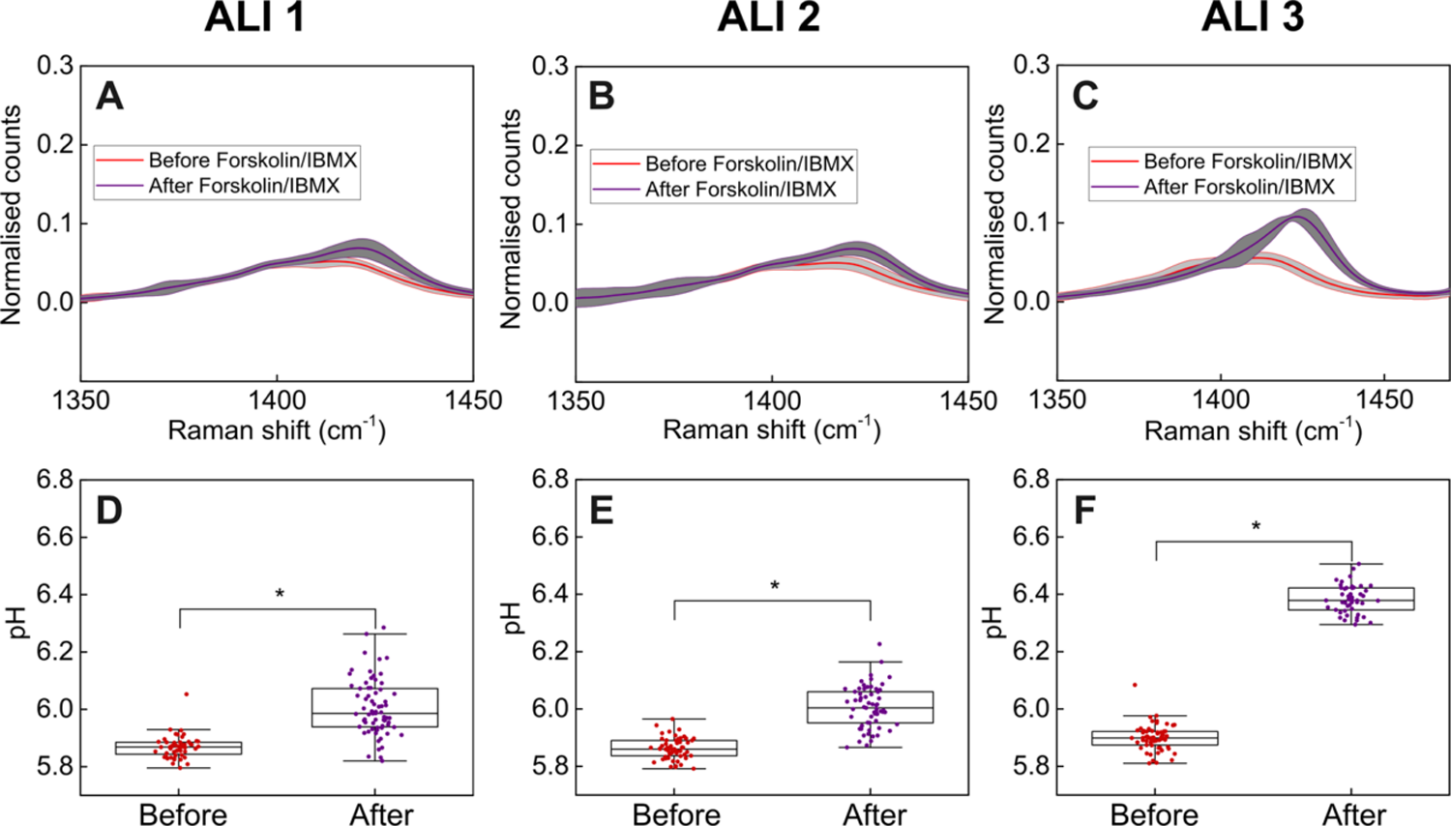
(A–C) Mean *v*_s_(COO) peak (spectra normalized to *v*(ref)) of SERS-MS in the airway surface liquid of three ALIs before and after treatment with forskolin/IBMX (complete spectra are presented in Figure S6). Each ALI was grown from a different healthy donor. Gray area indicates the standard deviation of the normalized spectra in each sample. (D–F) Airway surface liquid pH of ALIs before and after forskolin/IBMX treatment. Each data point represents the pH calculated from a single SERS-MS spectrum using the calibration curve in Figure 3. The center line of the box plot is the median, bottom and top of the box show the 25th and 75th quantiles, respectively, and whiskers extend 1.5 times the interquartile range; * indicates *p* < 0.05 by *t* test.

In non-Cystic Fibrosis airways, the ion channel CFTR is expressed on the apical surface of epithelial cells and secretes HCO_3_^–^ into the airway surface liquid. Airway epithelial cells of individuals with Cystic Fibrosis express mutated forms of CFTR, or exhibit decreased or no expression of CFTR. CFTR modulators are used to treat Cystic Fibrosis by increasing the functional activity of CFTR.^28^ Therefore, measuring the response of CFTR to stimulation offers a valuable measure in the drug discovery process. To determine whether SERS-MS could measure the response of CFTR to stimulation in a live ALI culture, we measured the apical pH in healthy ALIs treated with Forskolin/IBMX to elevate intracellular cyclic adenosine monophosphate (cAMP) and open CFTR.^29^Figure 5 presents the *v*_s_(COO) vibrational mode of SERS-MS spectra and associated pH, collected from the airway surface liquid of 3 healthy ALIs from different donors before and after exposure to Forskolin/IBMX.

All three ALIs tested showed a significant increase in airway surface liquid pH following treatment with Forskolin/IBMX. We noted that the impact of Forskolin/IBMX on pH was different for each donor. In ALI 1 and 2, airway surface liquid pH only increased ∼0.1 pH units, while in ALI 3, it increased by ∼0.5 pH units. We also ran a control experiment to confirm that pH changes were the result of CFTR activity following Forskolin/IBMX treatment and not the effects of DMSO (the solvent of the Forskolin/IBMX stock solutions) (Figure S7). In ALI 1 and 2, the control treatment did not result in a significant pH increase, but in ALI 3, the pH increased by ∼0.1. However, the pH increase from Forskolin/IBMX treatment in ALI 3 was still larger than that in the control, indicating that activating CFTR significantly raised airway surface liquid pH in all three ALIs. These results show how SERS-MS can be used to explore the impact CFTR activity has on airway surface liquid pH, validate SERS-MS as a sensor of airway pH in Cystic Fibrosis, and demonstrate its use in measuring the response to CFTR modulators.^30^

### pH in Cystic Fibrosis and Non-Cystic Fibrosis ALIs

CFTR mutations may reduce the transport of HCO_3_^–^ to the airway surface liquid, lower pH, and impair airway defense against invading pathogens in people with Cystic Fibrosis.^18^ However, some studies have reported no significant difference between the airway surface liquid pH of Cystic Fibrosis and non-Cystic Fibrosis ALIs.^13^ To understand how CFTR mutations affect pH, we grew ALIs from six different Cystic Fibrosis and non-Cystic Fibrosis donors and measured the airway surface liquid pH with SERS-MS.

**Figure 6:**
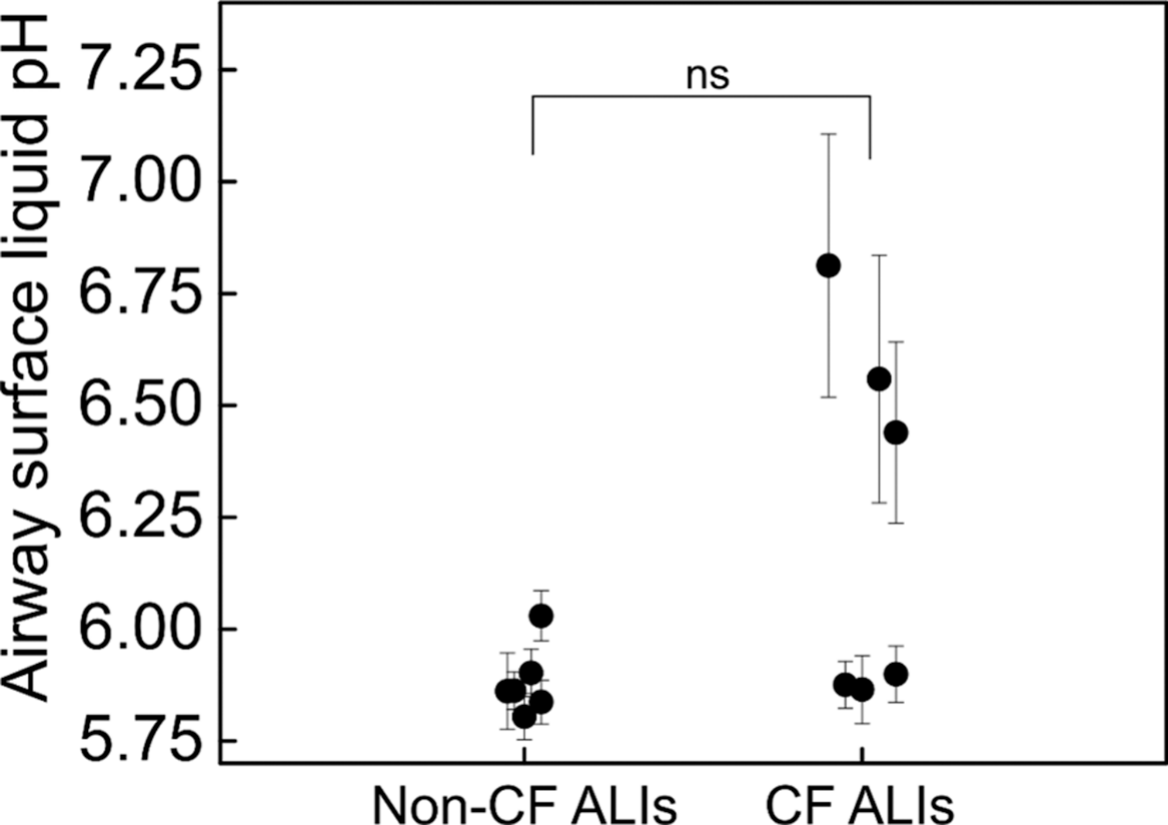
Airway surface liquid pH measured with SERS-MS in non-Cystic Fibrosis (non-CF) and Cystic Fibrosis (CF) ALIs. ALIs were grown from six different donors with and without Cystic Fibrosis. No significant difference in airway surface liquid pH was measured between non-Cystic Fibrosis and Cystic Fibrosis ALIs (*p* > 0.05 by Mann–Whitney *U* test), but Cystic Fibrosis ALIs showed greater pH variability (*p* < 0.05, by *F*-test).

The mean pH of Cystic Fibrosis ALIs was 6.2 ± 0.4, and in non-Cystic Fibrosis ALIs, it was 5.9 ± 0.1. While the difference between Cystic Fibrosis and non-Cystic Fibrosis pH was not statistically significant, we noted much more variability in the pH of Cystic Fibrosis samples. A two-sample test for variance (*F*-test) confirmed that the two population variances were significantly different (*p* < 0.05). This variability might be explained by the large phenotypic differences seen in patients with different CFTR mutations; each Cystic Fibrosis donor in this work had a different Cystic Fibrosis genotype (Table S4). More than 2000 CFTR disease-causing mutations have been identified and a combination of two mutated CFTR genes is required to cause the disease.^31^ Consequently, CFTR dysfunction can range from complete loss of channel function to less severe manifestations with residual channel function depending on mutation type.^32^ The more alkaline Cystic Fibrosis ALIs in Figure 6, with airway surface liquid pH 6.45–6.81, were from two samples with severe loss of function mutations and one with a residual function mutation. The more acidic Cystic Fibrosis group similarly comprised samples from a heterogeneous patient population, suggesting that pH may not completely reflect the effects of CFTR dysfunction, which is important if we consider that other epithelial cell ion channels may also contribute to apical pH. Therefore, while we demonstrate the principle that quantitative SERS measurements can be used to measure apical pH accurately between different donors, further studies focusing on other factors that might affect pH are required in tandem with larger studies of samples from people with Cystic Fibrosis with a range of CFTR mutations and consequent CFTR dysfunction.

## Conclusions

SERS-active microparticles (SERS-MS) can be employed to measure pH gradients and dynamic pH events in human-donor-derived respiratory epithelium models. Using SERS-MS, a pH gradient of ∼1 pH unit was measured between the airway surface liquid and the basolateral cell culture medium of Cystic Fibrosis ALIs demonstrating the utility of SERS to measure physiochemical gradients formed across epithelial junctions in a monolayer, which may in themselves be a measure of tight junction functionality at baseline. Our SERS-MS is sufficiently sensitive to measure airway surface liquid pH changes induced by activating CFTR in healthy ALIs. This demonstrated SERS-MS as a powerful tool to monitor the activity of the ion channel that causes Cystic Fibrosis when mutated and may find utility in screening for therapies that restore CFTR function. In ALIs grown from donors with and without Cystic Fibrosis, we noted greater pH variability in Cystic Fibrosis ALIs compared to healthy ALIs suggesting that apical pH may not simply be a surrogate measurement of CFTR function. Overall, we show that SERS-MS are versatile extracellular pH sensors that can provide real-time functional information in primary cell culture underlining the potential for the development of this technique in additional ex vivo culture models across a range of diseases and organ systems.

## Supporting Information Available

SERS-MS buffeted by beating cilia in airway surface liquid (MP4)
Culture medium formulation; equations; Cystic Fibrosis genotypes, and additional airway surface liquid pH measurements (PDF)
