# Supplementary material for: SERS Microsensors for the Study of pH Regulation in Cystic Fibrosis Patient-Derived Airway Cultures
Source: ACS Sens. 2024 Apr 25;9(5):2550–7. doi: 10.1021/acssensors.4c00279 (PMC11129347; doi:10.1021/acssensors.4c00279)
Supplement: Supplementary file 2 — se4c00279_si_002.pdf [file se4c00279_si_002.pdf]

SERS microsensors for the study of pH regulation in Cystic Fibrosis patient-derived airway cultures.

William H. Skinner,[a] Nicola Robinson,[b] Gareth R. Hardisty,[b] Robert D. Gray[b] and Colin J. Campbell[a]*

[a] EaStCHEM School of Chemistry, University of Edinburgh, The University of Edinburgh, King's Buildings, Mayfield Road, Edinburgh EH9 3FJ, United Kingdom

[b] Centre for Inflammation Research, The Queen's Medical Research Institute, 47 Little France Crescent, The University of Edinburgh, Edinburgh EH16 4TJ, UK.

Supporting information

Tables and equations

Table S1. Basal cell expansion medium formulation, with supplier and concentration

Solution	Supplier	Final volume or concentration in medium
Pneumacult™ Ex-Plus Medium	Stemcell	490 ml
Pneumacult™ Ex Plus 50x Supplement	Stemcell	1x
DMH-1	Tocris	200 nM
CHIR99021	Tocris	500 nM
A83-01	Tocris	500 nM
Y-27632	Abmole	5 mM
Amphoteracin B	Sigma	250 ng/ml
Gentamicin	Sigma	10 µg/ml
Penicillin / Streptomycin	Invitrogen	100 µg/ml / 100 mg/ml
Primocin	Invitrogen	50 mg/ml
Hydrocortisone	StemCell	96 ng/ml

Table S2. ALI medium formulation, supplier and concentration

**Solution**	**Supplier**	**Final volume or concentration in medium**
Pneumacult ALI base medium	StemCell	45ml
Pneumacult ALI 10x supplement	StemCell	1x
Pneumacult ALI Maintenance supplement	StemCell	500µL
Heparin 0.2%	StemCell	100µL
Hydrocortisone	StemCell	480 ng/ml
Penicillin / Streptomycin	Invitrogen	100 µg/ml / 100 mg/ml
Primocin	Invitrogen	50 mg/ml

Equation used to fit pH calibration curve.

$$\boldsymbol{Y} = \frac{(\boldsymbol{A} - \boldsymbol{B})}{1 + \boldsymbol{e}^{\frac{\boldsymbol{pH} - \boldsymbol{c}}{\boldsymbol{dx}}}} + \boldsymbol{B} \qquad \text{Equation S1}$$

Table S3. Value of each variable in Equation S1 following fitting to calibration data

**Variable**	**Value from fit**
$\boldsymbol{A}$	-0.31795
$\boldsymbol{B}$	0.22402
$\boldsymbol{c}$	5.19262
$\boldsymbol{dx}$	0.87901

Equation used to calculate TEER.

$$TEER = (\Omega_{ALI} - \Omega_{Blank}) \times Area_{Transwell} \qquad \text{Equation S2}$$

Table S4. Cystic Fibrosis mutation class and ASL pH measured with SERS-MS

Genotype	Mutation class	Mean ASL pH
delF508/Q493X	I	6.4
delF508/N1303K	II	5.9
delF508/delF508	II	6.8
delF508/G551D	III	5.9
delF508/R117H/5T/9T	IV	5.9
delF508/c.579+3A>G	V	6.6

Figures

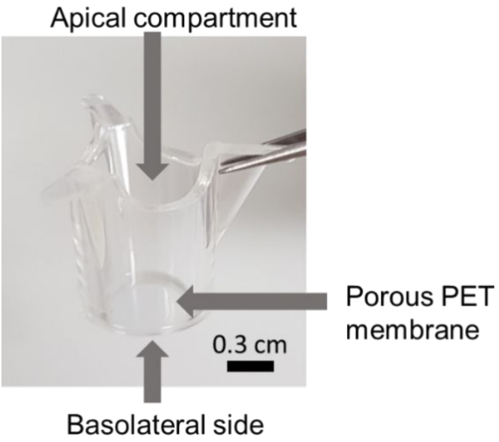

Figure S1. Image of 24-well Tissue Culture (TC) Insert (Sarstedt, 83.3932.041) with a pore size of 0.4 μm and a pore density of $2{\times}10^6$ pores/cm^2

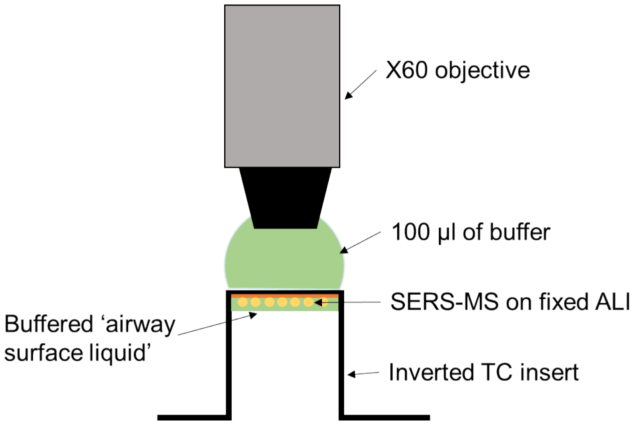

Figure S2. Schematic of experimental set-up used to collect SERS spectra from the apical surface of fixed ALIs for pH measurements.

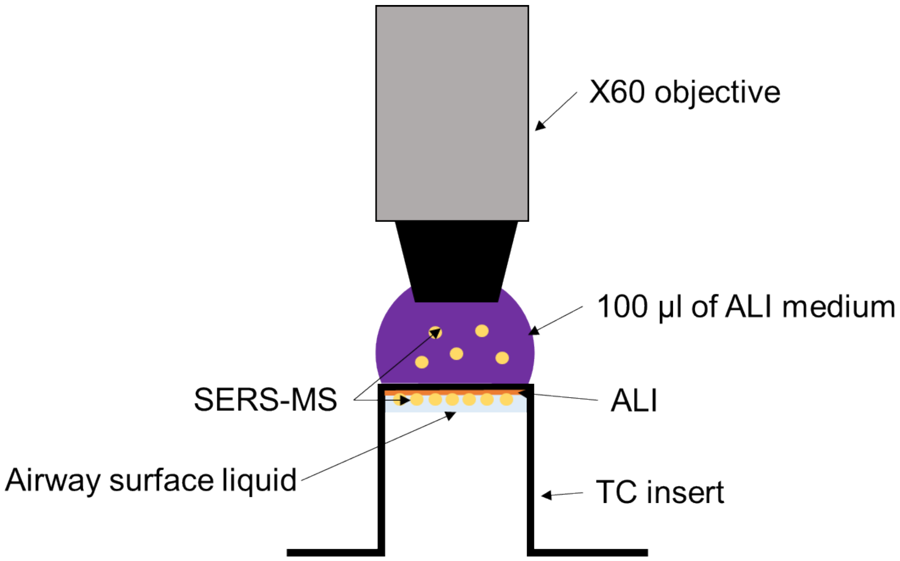

Figure S3. Schematic of experimental set-up used to measure pH in the airway surface liquid and the basolateral medium of ALIs.

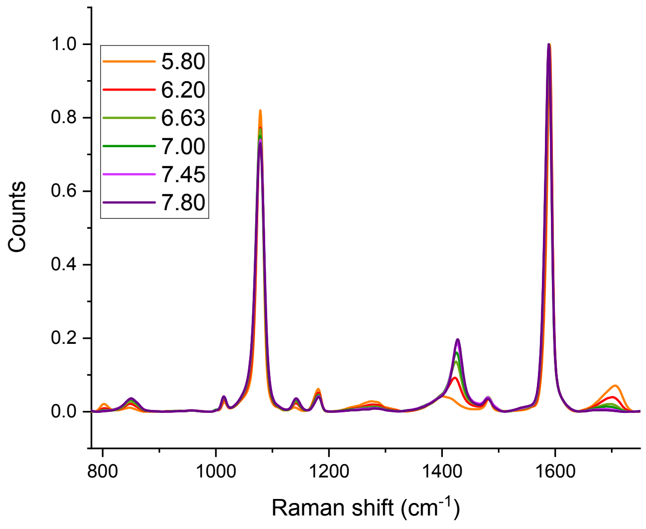

Figure S4. Mean SERS-MS spectra collected in pH adjusted cell culture medium. Spectra were normalised to the intensity of *v*(ref) prior to averaging.

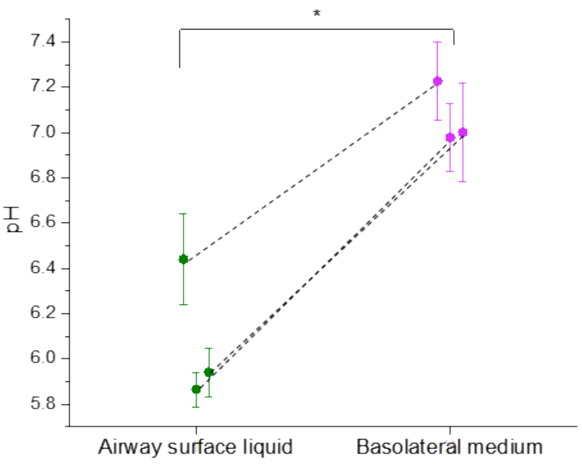

Figure S5. pH measured with SERS-MS in the airway surface liquid (apical) and basolateral compartment of 3 ALIs (including the ALI presented in Figure 4) grown from different donors with Cystic Fibrosis. Dotted lines link the apical and basolateral pH values measured in individual ALIs. * indicates there is a significant difference between the mean apical and basolateral pH measurement (paired t test comparing the mean pH value in the airway surface liquid and basolateral medium of each ALI, $p = 0.01$).

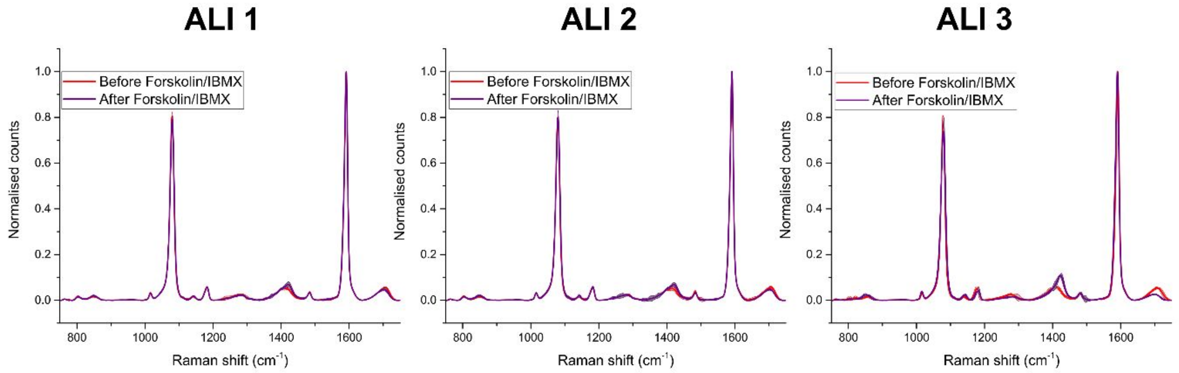

Figure S6. Mean spectra of SERS-MS (normalised to ν(ref)) in the airway surface liquid of three healthy ALIs before and after treatment with Forskolin/IBMX.

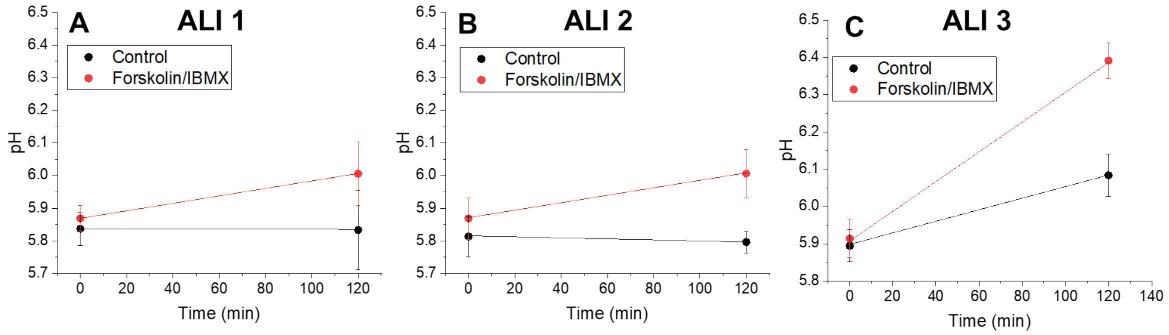

Figure S7. Airway surface liquid pH before (0 min) and after (120 min) the addition of Forskolin/IBMX or DMSO (control) to the basolateral cell culture medium. Data points represent the mean pH and error bars the standard deviation in spectral pH measurements. Lines link the 'before' and 'after' pH measurements in an individual ALI.
